# Mesenchymal Stem/Stromal Cells as a Therapeutic Tool in Cell-Based Therapy and Regenerative Medicine: An Introduction Expertise to the Topical Collection

**DOI:** 10.3390/cells11193158

**Published:** 2022-10-08

**Authors:** Makram Merimi, Hassan Fahmi, Joery De Kock, Charline Beguin, Arsène Burny, Guido Moll, Alessandro Poggi, Mehdi Najar

**Affiliations:** 1 LBBES Laboratory, Genetics and Immune Cell Therapy Unit, Faculty of Sciences, University Mohammed Premier, Oujda 60000, Morocco; 2 Department of Experimental Hematology, Jules Bordet Institute, Université Libre de Bruxelles, 1000 Brussels, Belgium; 3 Osteoarthritis Research Unit, Department of Medicine, University of Montreal Hospital Research Center (CRCHUM), Montreal, QC H2X 0A9, Canada; 4 Liver Therapy & Evolution Team, Research Group of In Vitro Toxicology and Dermato-Cosmetology, Faculty of Medicine and Pharmacy, Vrije Universiteit Brussel, 1090 Brussels, Belgium; 5 Laboratory of Immunophysiology, GIGA Institute, Liege University, 4000 Liège, Belgium; 6 Laboratory of Molecular and Cellular Biology, Faculté Universitaire des Sciences Agronomiques de Gembloux (FUSAGx), 5030 Gembloux, Belgium; 7 BIH Center for Regenerative Therapies (BCRT) and Berlin Brandenburg School of Regenerative Therapies (BSRT), Berlin Institute of Health (BIH), Charité—Universitätsmedizin Berlin, 10117 Berlin, Germany; 8 Molecular Oncology and Angiogenesis Unit, IRCCS Ospedale Policlinico San Martino, 16132 Genoa, Italy; 9 Department of Hematology, Jules Bordet Institute, Université Libre de Bruxelles, 1070 Brussels, Belgium

We are pleased to present this opening editorial, introducing our topical collection, “The New Era of Mesenchymal Stromal/Stem Cell Functional Application: State of the Art, Therapeutic Challenges and Future Directions”. This topical collection, edited by Prof./Dr. Alessandro Poggi and Dr. Mehdi Najar, belongs to the “Cellular Immunology” section of *Cells*. Under the direction of Dr. Mehdi Najar, a group of experts have contributed to this edition. We hope that this topical collection will contribute greatly to the field through its discussion and presentation of new outcomes linked to the functional application of mesenchymal stem/stromal cells in cell-based therapy and regenerative medicine.

Mesenchymal stromal/stromal cells (MSCs) are considered to be highly promising therapeutic products for various clinical applications with unmet clinical needs [1,2,3]. They have attracted great interest in the fields of immuno-therapy and regenerative medicine [4,5]. MSCs have several important biological functions, including the support of hematopoiesis, tissue repair and anti-inflammatory effects [5,6].

Residing in different niches, these cells are involved in tissue homeostasis, and they can be found in almost all vascularized tissues, a fact which facilitates their isolation and expansion [7,8,9]. Subpopulations of MSCs derived from different anatomical locations demonstrate heterogeneity regarding phenotype, plasticity and function [8,9,10]. Thus, the plasticity in regard to the phenotypes and functions of MSCs should be further investigated. Upon ex vivo/in vitro isolation, the cells display a fibroblast-like morphology with the ability to adhere to plastic.

Interestingly, MSCs are attracted and migrate/home to damaged tissue sites, where they can exert their beneficial effects through both direct local and indirect systemic mechanisms [5,11]. Within the injured site, MSCs can moderate immune and inflammatory responses and coordinate endogenous tissue repair. Although MSCs elicit a strong modulation of the immune system, they should not be considered as an immune cell, despite their complex interactions with the immune system [12]. Although MSCs are generally considered to be hypoimmunogenic, the immunogenicity of individual MSC products needs to be considered in advance [13] in order to avoid any unnecessary side effects linked to the activation of innate or adaptive immune responses [8,9,14]. 

Importantly, MSCs have the capacity to actively assess the local molecular and cellular environment and respond adequately [6]. MSCs exert their effects via both contact-dependent mechanisms and through their secretome and extracellular vesicles (EVs) via the release and transfer of active factors, including growth factors, cytokines and genetic material, to target cells and tissues [15]. The therapeutic cargo molecules can be stored within EVs, which are nano-sized membrane-bound vesicles that shuttle important signals between different types of cells and tissues in order to maintain physiological homeostasis [16]. By selectively isolating these MSC-derived vesicles, MSC-derived vesicles from a specific tissue may be infused, instead of the whole cell, for different clinical purposes. Interestingly, the immunosuppressive properties of MSC can be mediated by several cytokines and inhibitory factors that can be delivered to immune cells through the EVs. Importantly, the content of these MSC-derived EVs can be tailored to the required needs through the genetic manipulation and/or exogenous stimulation of the MSCs and their enrichment through the liposome delivery of cargo.

Understanding the mechanisms involved in the regenerative process governed by MSCs can help to open doors to new applications and perspectives in regard to their therapeutic use. While the capacity of MSCs to differentiate into local progenitors during tissue repair through therapeutic use in vivo is debatable and requires further characterization [5], novel methodologies for investigating the identity and heterogeneity of MSCs should be implemented [8,9]. It currently remains an open question as to how MSCs can be distinguished from, e.g., fibroblasts and pericytes, and how these cell types are related. Indeed, if the mechanisms influencing the plasticity of these cells were better understood, one could try to strengthen MSCs by applying specific licensing/priming signals [6]. The composition of the secretome underlying the paracrine pathway used by MSCs to mediate their effects may open the door to the use of cell-free therapeutic tools in regenerative medicine. On the other hand, both MSCs and their derived EVs can potentially also have detrimental effects on some diseases, such as neoplasia, cancer coagulopathy and various other thrombotic complications related to the presence of procoagulant cells and their released EVs in the blood stream [8,9,17]. Indeed, the function of these cells depends on the biological microenvironment in which they operate, and the EVs produced can differ from tissue to tissue. The strong crosstalk between MSCs and tumors can induce changes in both cell populations and, consequently, in their released EVs. Consequently, the use of EVs as cargo to deliver drugs to the tumor site may have pro- or anti-tumor effects, influencing the fate of tumor stem cells. This would suggest that a deeper understanding of the functional features of a specific phenotype of MSC is essential to enable the better selection of the MSCs used in different therapeutic settings, such as immune-oncology therapeutic strategies. 

Collectively, the aim of this topical collection is to present an innovative collection of research subjects, with an overview of the recent developments in the field of MSC-based therapy. 
